# Over-expression of transcription factor *ARK1* gene leads to down-regulation of lignin synthesis related genes in hybrid poplar ‘717’

**DOI:** 10.1038/s41598-020-65328-y

**Published:** 2020-05-22

**Authors:** Qinxia Ye, Xiaozhen Liu, Wen Bian, Zhiming Zhang, Hanyao Zhang

**Affiliations:** 1 Key Laboratory for Forest Resources Conservation and Utilization in the Southwest Mountains of China, Southwest Forestry University, Ministry of Education, Kunming, Yunnan 650224 China; 2 Key Laboratory of Biodiversity Conservation in Southwest China, State Forest Administration, Southwest Forestry University, Kunming, Yunnan Province China

**Keywords:** Plant genetics, Gene expression

## Abstract

Improving wood growth rate and wood quality are worthy goals in forest genetics and breeding research. The *ARK1* gene is one member of the *ARBORKNOX* family in all plants, which play an essential role in the process of plant growth and development, but the mechanism associated with its gene network regulation is poorly investigated. In order to generate over-expression transgenic hybrid poplar, the *agrobacterium*-mediated transformation was used to obtain transgenic hybrid poplar ‘717’ plants to provide insight into the function of the *ARK1* gene in poplar. Moreover, the morphology of transgenic plants was observed, and transcriptome analysis was performed to explore the *ARK1* gene function. The results showed that there were significant differences in pitch, stem diameter, petiole length, leaf width, leaf length and seedling height between *ARK1* transgenic seedlings and non-transgenic seedlings. The transgenic seedlings usually had multiple branches and slender leaves, with some leaves not being fully developed. The results of transcriptome analysis showed that the differentially expressed genes were involved in the growth of poplars, including proteins, transcription factors and protein kinases. Genes related to the positive regulation in plant hormone signal transduction pathways were up-regulated, and the genes related to lignin synthesis were down-regulated. The RT-qPCR analysis confirmed the expression levels of the genes involved in the plant hormone signal transduction pathways and phenylpropanoid pathway. In conclusion, the *ARK1* gene had a positive regulatory effect on plant growth, and the gene’s coding enzymes related to lignin synthesis were down-regulated.

## Introduction

As one of the most important renewable resources and industrial raw materials on the earth, wood has an important economic value. With the increase of the global population, the demand for wood is becoming increasingly prominent, so the research on wood formation is critical. Therefore, increasing both wood growth rate and improving wood quality has become a vital goal of forest genetics and breeding. The formation of wood is a very complex biological process, which is the result of the co-expression of many genes. It is the process of vascular cambium through continuous proliferation and differentiation to produce secondary xylem and outward secondary phloem^1^. The primary process of secondary plant growth is plant height growth and thickening growth^2^. Previous studies have shown that hormones, transcription factors and regulatory factors are involved in the process of regulating plant secondary growth^1,2^.

Signal transduction factors and transcription factors can receive signals and transmit to downstream transcription factors to regulate gene expression. Signal transduction and transcription factors play essential roles in the process of secondary growth. The signal transduction factors include auxin and mitogen, and the transcription factors involved in the regulation of secondary growth include the *WOX* gene^3,4^, *NAC* gene^5,6^, *MYB* gene and *ARBORKNOX* gene^7–10^.

The *ARBORKNOX* gene family is one of the homeobox gene families in plants, which exist in almost all plants and play essential roles in the process of plant growth and development^10–13^. *ARBORKNOX* genes are directly involved in the regulation of plant lignification and cell wall synthesis^10,14^. *KNOX* homologous transcription factors regulate plant growth and development in a variety of ways, interact with hormone pathways mediated by auxin, gibberellin (GA) and mitogen (CK) to activate signal pathways in plants, and have been shown to regulate the genes encoding GA biosynthesis directly^11,12^. GA has been reported to affect the lignification of stem cell walls^8^.

Advances in molecular biology provide new methods to study secondary growth and cambium function in forest trees. There are many economically and ecologically important species in the genus Populus, with some developed as popular models for molecular biology in angiosperm trees. The Populus genome has been sequenced and would be exceedingly useful in the study of secondary growth and cambium function. The creation of gene over-expression stable lines is widely used in protein engineering, drug discovery, gene functional analysis, and other basic researches. RNA-Seq, a deep-sequencing technology, is a useful method for transcriptome profiling. Studies using this approach have already expanded our view on the complexity of poplar transcriptomes. Yao *et al*. (2018) used RNA-Seq to screen differentially expressed genes (DEGs) and detect the *NAC* family in poplar leaves^15^. In an analysis of the overexpression of *AtGolS3* and *CsRFS* in poplar, La Mantia *et al*. (2018) found that transcriptome analysis and qRT-PCR validation revealed the genetic network of the defence response to poplar leaf rust disease^16^. After transcriptome analysis of *MYB165-* and *MYB194-*overexpressing poplars, Ma *et al*. (2018) found that *MYB165* and *MYB194* were negatively related to many phenylpropanoid enzyme genes and shikimate pathway enzyme genes^17^.

This paper uses transgenic technology and high flux sequencing techniques to sequence and analyse the transcriptome of transgenic and non-transgenic hybrid poplar ‘717’. This technology is adopted to identify critical metabolic pathways and genes involved in poplar secondary growth, explore the effect of transcription factor *ARK1* on secondary growth of woody plants and study the functions of secondary growth-related genes in poplar. Moreover, the functional annotation, functional classification and metabolic pathway enrichment of the differentially expressed genes (DEGs) were studied.

## Materials and methods

### Plant and bacterial materials

The explants used in the experiment were taken from the hybrid poplar ‘717’ (INRA 717-1B4, a female *P.tremula* × *P.alba*) grown in Southwest Forestry University, Kunming, China. The young leaves and stem segments of hydroponics and root sprouts were selected as explants. Shanxi Bored Biotechnology Co. Ltd. (Shanxi, China) synthesised the *ARK1* gene, which was then constructed into a binary vector, pCAMBIA 1300. *Agrobacterium tumefaciens* strain LBA4404 was preserved in our laboratory.

### Transformation

The leaves of hybrid poplar ‘717’ infected by *A. tumefaciens* were inoculated on a callus induction medium (MS + 1.0 mg/L NAA + 1.0 mg/L 6-BA) and co-cultured at 28 °C for 2–4 days. The co-cultured calli were washed with aseptic water three times, dried with aseptic paper, and then transferred to an aseptic differentiation medium (MS + 1.0 mg/L 6-BA + 0.4 mg/L ZT) containing carbenicillin and kanamycin. The selective culture was carried out under 8 to 16-hour photoperiod conditions at 28 °C. Approximately 28 days later, the medium was changed to induce new calli to form and sprout. When the adventitious buds grew to 2 to 3 cm, they were moved to a rooting medium (1/2 MS + 0.02 mg/L NAA + 0.6 mg/L IBA) containing carbenicillin and kanamycin for root culture. When the adventitious roots grew to 2 to 3 cm, the plants were moved to a greenhouse.

### Identification of transformants

#### PCR analysis

The DNA was extracted with an HiPure SF plant DNA mini kit (Magen Company, New York, USA) from transgenic and non-transgenic seedlings. The sequences of the primers were 5′-AAGATCCAGCCCTTGACCAA-3′ and 5′-CATTGCCATCACCACAACCA-3′. Then the PCR reaction was carried out in a GeneAmp RCR System 9600 (Perkin Elmer, Foster City, CA, USA) under the PCR conditions of 94 °C for 3 min; 94 °C for 30 sec, 55 °C for 30 sec, 72 °C for 5 min, 35 cycles; 72 °C for 10 min.

#### Measurement of morphological changes in transgenic plants

The morphological differences of tissue-cultured seedlings between three different transgenic lines with a specific PCR band and three non-transgenic plants that underwent the same conditions were compared. The diameter of stem segments, the number of internodes and the length of internodes at the same growth stage were measured.

### Transcriptome analysis

The experimental materials were taken from the stem segments under the fifth leaf to the sixth leaf of three transgenic lines and three non-transgenic seedlings. The sampling time was at 11: 00 am.

The purified samples were sequenced using the HiSeq high-throughput sequencing platform by Shanxi Bored Biotechnology Co., Ltd. (Shanxi Province, China). The genome of hybrid poplar ‘717’ was used as the reference genome, and the download address was http://aspendb.uga.edu/index.php/databases/spta-717-genome.

DESeq was used to analyse the differential expression among different groups^18^. The DEGs between the two biological conditions were obtained, and the DEGs were classified. Then the phyper function of R software was used for enrichment analysis. Those DEGs with fold change ≥2 and FDR < 0.01 were regarded as significant enrichment.

Additionally, a DEG pathway annotation analysis was used to analyse the functions of the genes further. Those DEGs with fold change ≥2 and FDR < 0.01 were regarded as significant.

### RT-qPCR analysis

This study selected the transcriptomic expression levels of ten genes including the *ARK1* gene and nine genes involved in the plant hormone signal transduction pathways and the phenylpropanoid pathway for validation by RT-qPCR analysis in three transgenic lines and three non-transgenic plants used in the transcriptome analysis. The elongation factor gene *EF1β* was used as an internal control^19^. The total RNA was extracted using the Qiagen RNeasy Mini Kit (Qiagen Inc., Valencia, CA), and then reversely transcribed into cDNA by random primers. The RT-qPCR analysis was conducted according to a previous report^20^. Gene-specific primers were designed using Primer Premier 5.0 software, and Table 1 lists the sequences of primers used for RT-qPCR analyses. The 2^(−ΔΔCt)^ method was used to analyse the data^20^.

**Table 1:** Primer sequences of target genes (TG) and reference gene (RG) used in RT-qPCR.

Gene name	Primer	Sequence (5′-3′)	Length (bp)
Elongation factor *EF1β* (RG)	Forward	GACAAGAAGGCAGCGGAGGAGAG	269
	Reverse	CAATGAGGGAATCCACTGACACAAG	
*ARK1*	Forward	CATCCATCACCACAAACTGC	165
	Reverse	ATTGGTGGAGCAGGCATTAC	
Cyclin-D3-1 protein	Forward	GGCGTAATTGGTGCTGTTTT	155
	Reverse	AGATGAAGGTGGGCTGCTAA	
PYR1 abscisic acid receptor	Forward	CACACCTACAGAATCAGCGC	166
	Reverse	CCGACGGTCATTGTGAATCC	
Histidine phosphate transfer protein	Forward	GAAAGTCTGCATTGGCTTCC	219
	Reverse	TTTTGCTGTTTCTGGCTGTG	
Indole-3-acetic amido synthetase	Forward	TCGAGTGGGGGACATACTTC	149
	Reverse	TGGTTAGCTGCATTCTGCAC	
Indole-3-acetic amido synthase GH3.6	Forward	TGGCTAGGCCAGTTCTCACT	194
	Reverse	CAGAAGCAAAAACAGCACCA	
Indole-3-acetic amido synthase GH3.9	Forward	ATGAGGGCAAAGCAATGTTC	184
	Reverse	GCTCTGGTTGCTATCCTTGC	
Trans cinnamic acid-hydroxylase	Forward	CCCTGTCTCGTACCCAATGT	246
	Reverse	CACCATGTCTTGTCCATTGC	
Peroxidase 17	Forward	CTTGCAGCCTTTCTCTTGCT	155
	Reverse	GGCAGCACTTCTTGGTTCTC	
Peroxidase 73	Forward	GGGCCTTCATATCCTGTTGA	184
	Reverse	TGAGAGAATCCAAGCGTGTG	

### Statistical analysis of data

Excel 2007 (Microsoft, Redmond, WA) and SPSS 18.0 were applied to the statistical analysis and mean ± *standard errors* (SEs) were calculated.

## Results

### Transformants of *ARK1*

Thirty-five seedlings that grew on an MS medium containing carbenicillin and kanamycin were selected for PCR amplification. Eleven seedlings with a transformation marker were identified. About 31.43% of plants were PCR positive. The RT-qPCR analysis results showed that the expression levels of the *ARK1* gene in transgenic lines were about 2.95 times that of control seedlings (*p*-value = 0.000028, significant at *p* < 0.01).

### Comparison of the growth between *ARK1* transgenic and non-transgenic seedlings

**Figure 1:**
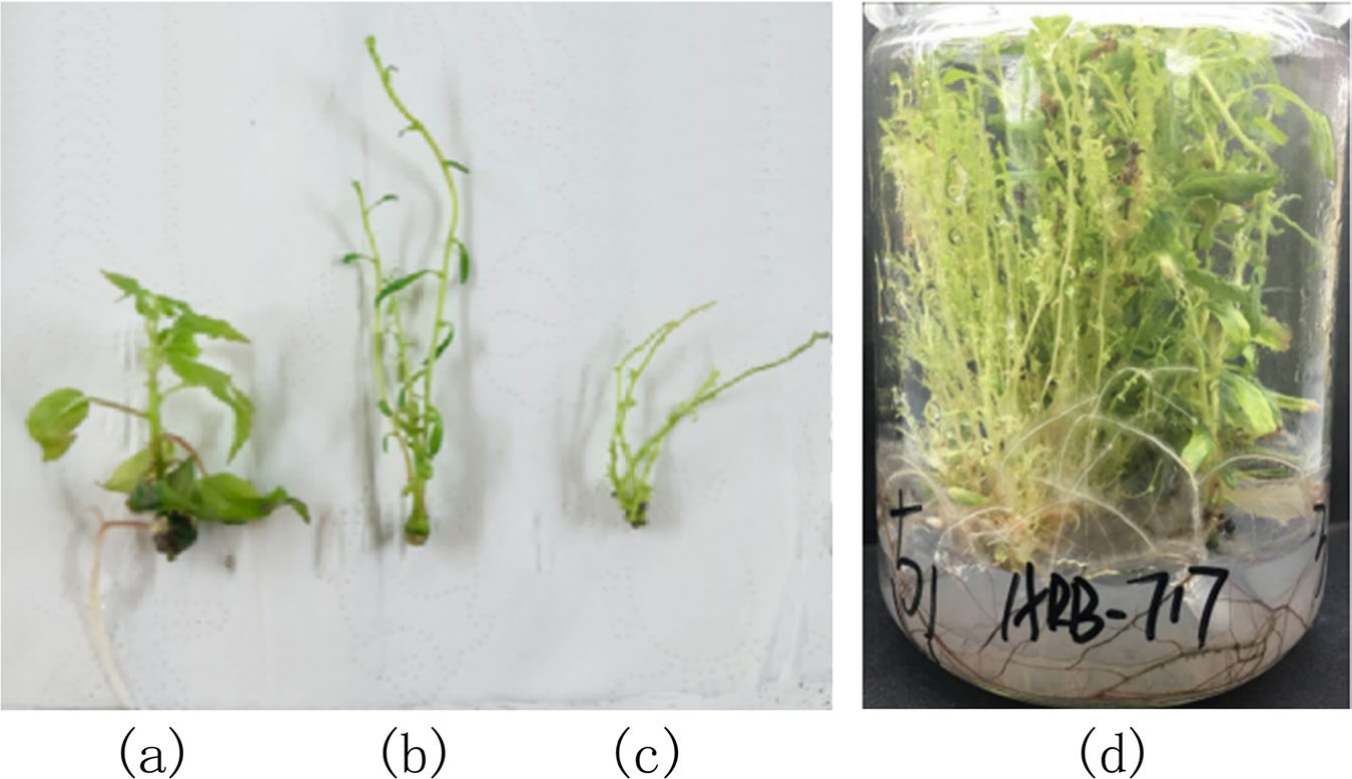
Non-transgenic and Transgenic seedlings. (**a)** Non-transgenic transgenic plant; (**b**) *ARK1* transgenic plant with slender stem segments and slender leaves; (**c**) *ARK1* transgenic plant with slender stem segments and undeveloped leaves; (**d**) *ARK1* transgenic plant in a tissue culture medium.

The transgenic and non-transgenic seedlings were weighted at the same growth stage (45 days). Compared to non-transgenic plants, transgenic plants had slender stem segments (usually fasciculated and multi-branched), slender leaves and undeveloped leaves (Fig. 1). The appearance of *ARK1* transgenic seedlings was consistent with the ones obtained by Groover *et al*.^21^. There were significant differences between transgenic and non-transgenic seedlings in node space, stem segment diameter and leaf width. The internode distances, stem segment diameters, and width and length of leaves were measured in the fifth and sixth leaves. There were significant differences in node spacing, stem diameter, petiole length, leaf width, leaf length and seedling height between transgenic and non-transgenic seedlings (Table 2).

**Table 2:** Growth data related to transgenic and non-transgenic seedlings.

Appraisal index (mm)	Non-transgenic seedlings	Transgenic seedlings	P-value
Internode distance	6.19 ± 0.12	11.86 ± 0.09	0.043*
Stem diameter	1.29 ± 0.07	0.72 ± 0.08	0.005**
Petiole length	12.87 ± 0.23	0.53 ± 0.03	0.000**
Leaf width	5.62 ± 0.18	1.09 ± 0.04	0.001**
Leaf length	14.09 ± 0.78	3.42 ± 0.11	0.009**
Seedling height	48.53 ± 1.56	76.27 ± 1.13	0.050*

**Indicated that the difference was extremely significant, and * indicated that the difference was significant (*P-value < 0.05, **P-value < 0.01).

### Analysis of transcriptome data

After the original data were filtered by quality control, the redundant sequences and low-quality reads were removed, and a total of 45.8 GB clean reads were obtained. The percentage of Q30 bases was higher than 91.85%, and the average GC content of the six samples was 47.69%, indicating that the sequence quality was good and met the requirements of database construction. Sequences were aligned between the clean reads and the reference genome of *P. tomentosa*, and alignment efficiency varied from 55.61% to 60.61%. Reference genomes could annotate approximately 57.53% of the sequences. The clean reads alignment rate of the reference sequence was 56.61%.

The total mapping ratio of the two groups compared with the previous reference genome was 58.81%. The lowest was 55.61%, and the highest was 60.61%. The average clean reads ratio of the two groups at a specific position of the reference genome was 74.11%, and the unique alignment between the groups was uniform, with the lowest at 73.46% and the highest at 74.86%. The comparison results showed that the comparison efficiency between the reads of each group and the reference genome ranged from 55.61% to 60.61%, and the selected reference genome met the requirement for analysis. A total of 641 DEGs were identified, of which 389 were up-regulated, and 252 were down-regulated.

**Figure 2:**
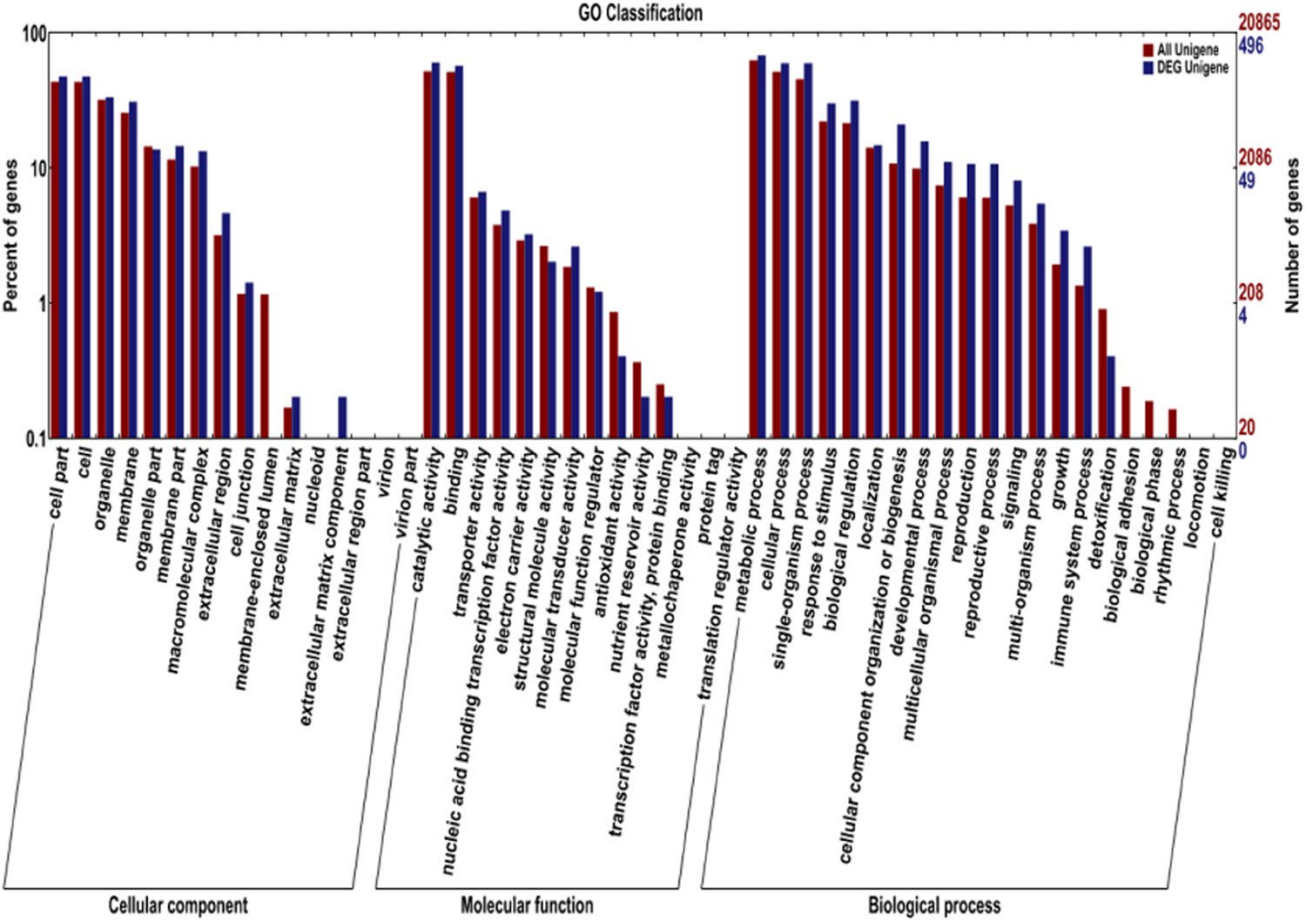
GO secondary node annotation statistics of differentially expressed genes. Transverse coordinate, the GO classification; the number at the left side of the vertical coordinate, the percentage of the number of genes; the number at the right side, the number of genes.

Through Gene Ontology (GO) enrichment analysis of 641 common differentially expressed genes in transgenic and non-transgenic plantlets (Fig. 2), a total of 496 DEGs were obtained in the enrichment entry of GO, and 428 DEGs belonged to the biological process, of which 237 were up-regulated, and 191 were down-regulated. Three hundred and eight DEGs belonged to the cell composition, of which 201 were up-regulated, and 107 were down-regulated. Moreover, 426 DEGs belonged to molecular function, of which 236 were up-regulated, and 190 were down-regulated. In the biological process category, there were 31 DEGs involved in protein phosphorylation, followed by 25 DEGs in the cellular metabolic process. Nineteen DEGs belonged to cell proliferation. Regarding cell locations, most DEGs were in the nucleus with 58 DEGs, followed by an integral component of the membrane, and the whole component of the membrane. There were 49 DEGs in the plasma membrane. In the molecular function category, most DEGs were ATP binding, followed by DNA binding and transcription factor activity. Furthermore, 24 DEGs belonged to sequence-specific deoxyribonucleic-acid-binding, including transcription factor activity, sequence-specific DNA binding and microtubule-binding.

### Functional analysis of differentially expressed genes

**Figure 3:**
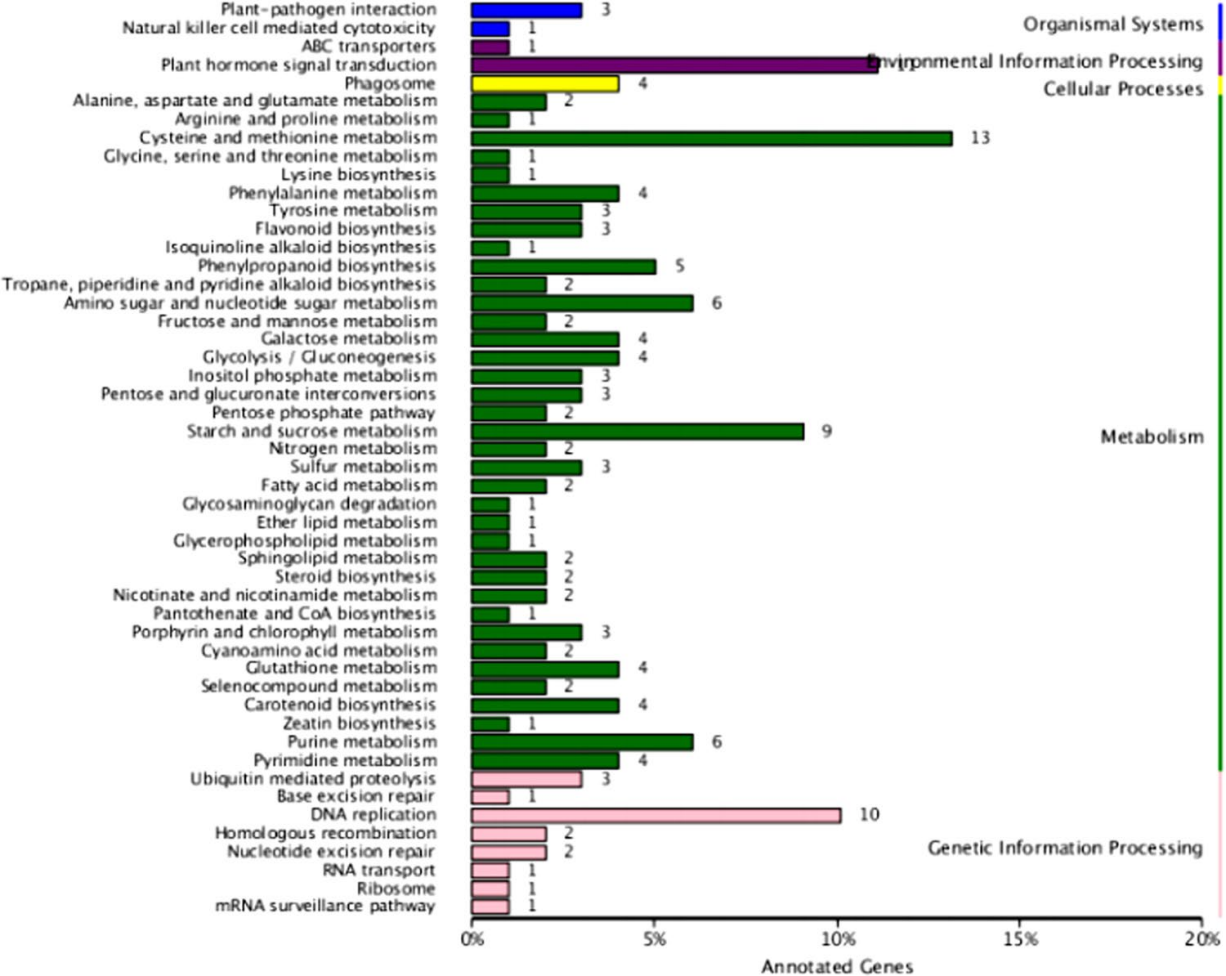
KEGG classification map of differentially expressed genes. The vertical coordinate, the name of the KEGG metabolic pathway; the transverse coordinate, the number of genes annotated to the pathway and the proportion of the number of genes in the total number of genes annotated.

Next, pathway enrichment analysis of 641 DEGs was carried out using the KEGG database. Metabolic pathways annotated were the organic system, environmental information processing, cell processing, metabolic and genetic information processing (Fig. 3). In this enrichment process, the metabolic pathway was the most significantly enriched pathway with 112 DEGs, followed by genetic information processing with 21 DEGs.

Results of annotation comparison showed that 160 significantly different genes in the hybrid poplar ‘717’ were annotated and enriched into 57 metabolic pathways. Thirteen differentially expressed genes were expressed in the metabolic pathway (KO: ko01100) pathway, and 11 DEGs were enriched in the plant hormone signal transduction (KO: ko04075) pathway. There were ten DEGs in the DNA replication (KO: ko03030) pathway and nine DEGs in the starch and sucrose metabolism (KO: ko00500) pathway. There were six DEGs in the amino sugar and nucleotides glucose metabolism (KO: ko00520) pathway and six DEGs in the purine metabolism (KO: ko00230) pathway.

### Screening and analysis of growth-related DEGs

GO enrichment analysis and pathway functional annotation was applied to screen the expression and regulation of DEGs related to the secondary growth. The cell tip growth pathway (GO:0009932), meristem growth pathway (GO:0010075), plant hormone signal transduction (Ko04075) and phenylpropanoid biosynthesis (Ko00940) were selected for analysis. There were six DEGs related to cell tip growth, five DEGs related to meristem growth regulation, 11 DEGs related to plant hormone signal transduction pathways, and five DEGs involved in the phenylpropanoid biosynthesis pathway. Furthermore, genes were annotated using the NCBI database (Table 3).

**Table 3:** Annotation of growth-related differentially expressed genes.

GIDs	NCBI annotation	log2FC	Gene ID
XM_006368111.2	Oxidase-like protein	1.65080893942576	18093985
XM_002323811.3	ABC transporter B family	2.04634861811701	7495965
XM_006369586.2	Tubulin β-4 chain	1.65080893942576	18095120
XM_002322573.3	Tubulin β-5 chain	1.65080893942576	7464075
XM_002307655.3	WALLS ARE THIN 1 protein	1.59553464943038	7477644
XM_006368330.2	TORNADO 2 protein	3.25535317419646	18094158
XM_002310565.3	Cytochrome P450 78A7	5.00597636395377	7456964
XM_002310515.3	Peroxidase 17	−1.3784859676881	7466794
XM_002304181.2	Homeobox-leucine zipper protein	2.35676034864413	7478415
XM_002298707.3	Cyclin-D3-1 protein	2.33367747592877	7492059
XM_002311147.3	Phosphatase 2C 37	−1.31626762054384	7467562
XM_002318999.2	Phosphate transfer protein containing histidine	1.64126125792861	7473313
XM_002319362.2	Indole-3-acetic amide synthetase	3.18842694097824	7497419
XM_006370518.2	Indole-3-acetic amide synthetase GH3.6	3.21975623148009	18095866
XM_006385591.2	PYR1 abscisic acid receptor	4.46474025247368	18096970
XM_024581841.1	Phosphatase protein 2C 51	−2.70201706614217	7489826
XM_024591467.1	EIN4-like protein	−2.70201706614217	112325302
XM_024594626.1	Indole-3-acetic amide synthetase GH3.9	4.16778849064521	7478832
XM_024607193.1	Ethylene response transcription factor 1B	−4.29034119395611	18101603
XM_024610415.1	Ethylene receptor	−2.46226108301277	7498022
XM_002304914.3	β-glucosidase 46	5.05050992570199	7458571
XM_024598534.1	β-glucosidase 46	5.05050992570199	18097400
XM_024590232.1	Trans cinnamic acid-hydroxygenase	−2.08868270819994	112325022
XM_002310515.3	Peroxidase 17	−2.0507445279602	7466794
XM_024605163.1	Peroxidase 73	−2.0529527985568	18101129

**Figure 4:**
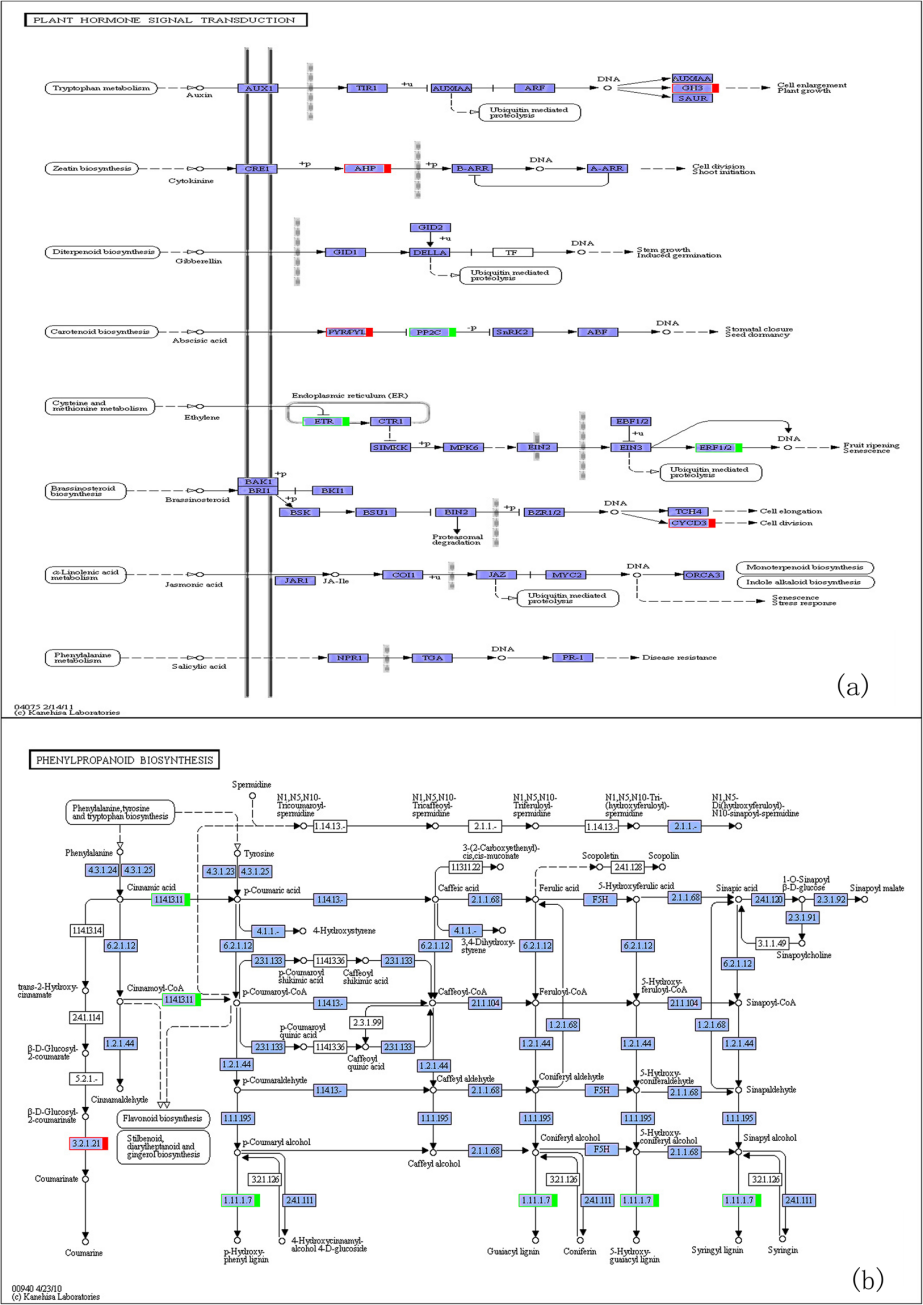
Pathways of the plant hormone signal transduction and the phenylpropanoid biosynthesis. (**a**) Pathways of plant hormone signal transduction; (**b**) Pathways of the phenylpropanoid biosynthesis. Red box, up-regulated genes; green box, down-regulated genes; blue box, both. (**a**,**b**) were KEGG pathway Maps^44^.

In the plant hormone signal transduction pathways (Fig. 4a), the following expression levels increased: XM_002298707.3 (cyclin-D3-1 protein, 2.33367747592877), XM_006385591.2 (PYR1 abscisic acid receptor, 4.46474025247368), XM_002318999.2 (histidine phosphate transfer protein, 1.64126125792861), XM_002319362.2 (indole-3-acetic amido synthetase, 3.18842694097824), XM_006370518.2 (indole-3-acetic amido synthase GH3.6, 3.21975623148009) and XM_024594626.1 (indole-3-acetic amido synthase GH3.9, 4.16778849064521). Cyclin-D3-1 protein, a cell cycle regulator, can regulate the expression of cyclin-dependent kinase (CDKs) in controlling the late and early S phase of the cell cycle. The *PYR1* abscisic acid receptor, a kind of receptor involved in signal transduction, plays an important role in plant growth and development. The phosphate transfer protein that contains histidine acts as a mitogen sensor and a two-component phosphorylation medium between histidine kinase and reaction regulator BARR. Indole-3-acetic amide synthetase, a GH3 auxin response promoter, was reported to participate in plant auxin signal transduction and play an important role in regulating growth and development^22^.

The genes involved in the plant hormone signal transduction pathways, including XM_024591467.1 (EIN4-like protein), XM_024610415.1 (ethylene receptor), XM_024607193.1 (ethylene response transcription factor 1B), XM_002311147.3 (phosphatase 2 C) and XM_024581841.1 (phosphate protease 2 C 51), were down-regulated. EIN4-like protein and ethylene receptors play a role in two-component system-based signal transductions in plant growth and development, acting as negative regulators of ethylene signal transduction in plant embryos, etiolated seedlings, leaves, roots, inflorescences and stamens. EIN4-like protein was expressed in pollen and tapetum and moderately expressed in carpels^23^. Ethylene response transcription factor 1B was reported to play an important part in plant growth and development and organ formation^24^. Phosphatase 2C 37 and phosphate protease 2C 51 both catalyse the dephosphorylation of phosphate serine and threonine phosphate residues of specific protein substrates, which can regulate the reversible phosphorylation of proteins in a variety of signal transduction pathways. Phosphatase plays a major role in plant growth and development and is mainly involved in the development of plant ears^25,26^. *PYR1* abscisic acid receptor proteins are receptors involved in signal transduction. They bind to abscisic acid (ABA) and mediate its signal transduction. After binding to ABA, these proteins interact with 2C protein phosphatase and inhibit its activity^27^.

There were both positive and negative regulatory genes among these growth-related genes screened. Up-regulated DEGs were mainly concerned with positive regulation, while the down-regulated DEGs were mainly concerned with negative regulation in the transgenic seedlings. These results indicated that the *ARK1* gene had a positive regulatory effect on plant growth.

**Figure 5:**
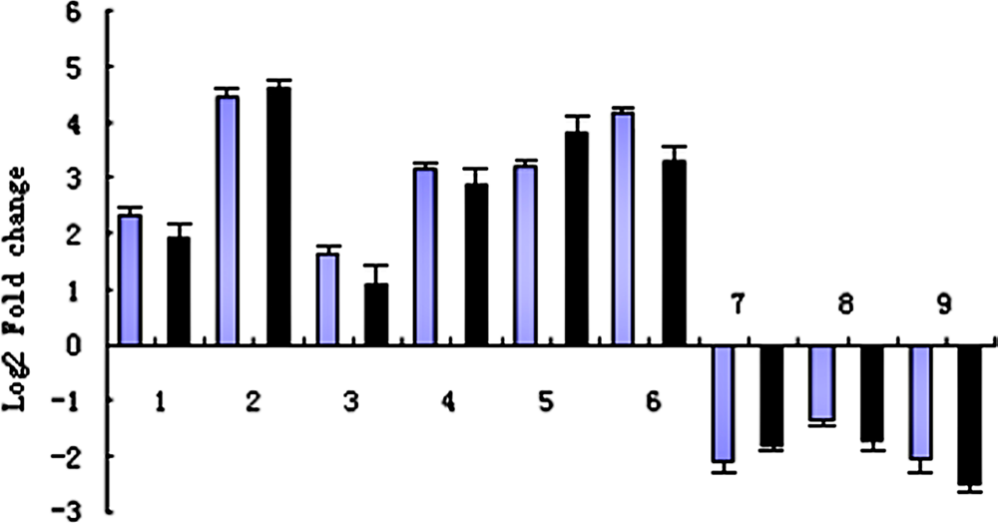
Comparison of RNA-sequencing and RT-qPCR results of selected DEGs. , RNA-seq; ◼, RT-qPCR. 1, Cyclin-D3-1 protein; 2, PYR1 abscisic acid receptor; 3, Histidine phosphate transfer protein; 4, Indole-3-acetic amido synthetase; 5, Indole-3-acetic amido synthase GH3.6; 6, indole-3-acetic amido synthase GH3.9; 7, Trans cinnamic acid-hydroxylase; 8, Peroxidase 17; 9, Peroxidase 73. RT-qPCR was performed on 3 transgenic lines used in the transcriptome analysis and 3 control seedlings, normalized with housekeeping gene EF1β, repeated 3 times. DEG, differentially expressed gene; RT-qPCR, Real time quantitative polymerase chain reaction.

The genes with increased expression in transgenic plants in the phenylpropanoid biosynthesis pathway (Fig. 4b) included XM_002304914.3 (β-glucosidase, 5.05050992570199) and XM_024598534.1 (β-glucosidase, 3.0702239239688). β-glucosidase, a glycosyl hydrolase, participates in carbohydrate transport and metabolism, including plant morphogenesis and energy metabolism, and plays an essential role in plant development^28,29^. The genes with decreased expression included XM_024590232.1 (trans-cinnamic acid-hydroxylase, -2.08868270819994), XM_002310515.3 (peroxidase 17, -1.3784859676881) and XM_024605163.1 (peroxidase 73, -2.0529527985568). Trans-cinnamic acid-hydroxylase was found to catalyse the formation of P-coumaric acid and p-coumaroyl-CoA (Fig. 5). Moreover, the phenylpropanoid pathway was reported to provide a variety of secondary metabolites in plants, participate in plant tissue differentiation and protect plant tissue from environmental stress^30,31^. Peroxidase belongs to class III of the heme-dependent peroxidase superfamily in plants. All members of the superfamily shared heme-repair groups and catalysed multistep oxidation involving hydrogen peroxide as electron receptors^32^. Peroxidases catalyse the removal of H_2_O_2_ and are involved in the oxidation of toxic reductants, lignin biosynthesis and degradation of thrombus, catabolism of auxin and responses to environmental stresses, such as injury, pathogen attack and oxidative stress^33^. The down-regulated genes – peroxidase 17 and peroxidase 73 – were found to catalyse the formation of p-hydroxyphenyl lignin, guaiacyl lignin, 5-hydroxyguaiacyl lignin and lilac lignin (Fig. 5).

### RT-qPCR validation

The RT-qPCR analysis results showed that the expression levels of all the selected genes were consistent with the transcriptomic analysis results (Fig. 5). The findings confirmed the increased expression levels of the positive regulatory genes in the plant hormone signal transduction pathways. Additionally, results showed that the genes coding enzymes related to lignin synthesis in the phenylpropanoid pathway were down-regulated in transgenic plantlets compared to non-transgenic plantlets.

## Discussion

Foreign gene transformation mediated by *A. tumefaciens* is the result of the interaction between bacteria and plant cells, which can usually affect the infection ability of *A. tumefaciens*. All the factors that can affect the infection ability of *A. tumefaciens*, the ability of plant cell transformation response and the ability of transformant regeneration will affect the transformation effect. Confalonieri *et al*. (2003) found that the transformation rate of poplar × *P. tomentosa* backcross hybrid was 1.22% and 2.59% respectively, which was easier to transform than that of the *P. tomentosa* male plant – the transformation rate of 1,319 male plants was only 0.34%^34^. This experiment obtained 35 candidates of transformed seedlings of hybrid poplar ‘717’. Overall, a total of 11 seedlings were positive in PCR detection, and the transformation rate was 31.43%.

In this study, secondary growth-related gene *ARK1* was transformed into hybrid poplar ‘717’ mediated by *A. tumefaciens*. There were significant differences in node spacing, stem diameter, petiole length, leaf width, leaf length and seedling height between *ARK1* transgenic and non-transgenic seedlings. Similar to this study, it was reported that the hybrid poplar with *ARK1* over-expression grew vigorously, and the branching ability was very strong, which was usually characterised by multiple branches in a single node^12^. Chuck *et al*. found that the genes in the *KNOX* gene family in *Arabidopsis thaliana* were expressed in stem apical meristem rather than in mature organs^35^. Over-expression of *KNOTTED1* (*KN1*) gene in transformed seedlings could change normal leaves into lobed leaves, jag from the base of the leaves, and display leaves that do not fully develop or expand or are without slender petioles. In this study, leaves that were not fully developed or expanded also appeared in the *ARK1* transgenic poplar lines, and the specific function of secondary growth-related genes could be changed through the abnormal expression of *transcription factors* (*ARK1*).

*ARK1* (*ARBORKNOX1*) and *ARK2* (*ARBORKNOX2*) genes are poplar homologous genes of *Arabidopsis STM* and *BP* (*BREVIPEDICELLUS*), which play an essential role in regulating cambium cell differentiation^11,21^. *ARK1* was widely expressed in the apical meristem (SAM) and vascular cambium region, and down-regulated in the terminal differentiation cells of the leaves and secondary vascular tissues of the apical meristem. Groover *et al*. (2006) were the first to clone the homologous gene *ARK1* of Arabidopsis *STM* in *Populus tomentosa*^21^ and found that *ARK1* was mainly expressed in the cambium. Over-expression of *ARK1* or *STM* was reported to inhibit the differentiation of xylem and phloem fibres, inhibit leaf development and shorten the length of internodes^21^. Combined with the expression analysis of *ARK1* in the process of adventitious bud and adventitious root formation, it was found that *ARK1* was mainly involved in primordium formation and further differentiation of meristem cells in the late primordium^36^. *ARK1* was also found to play a role in the differentiation of different meristem in the stem tip, root tip and formation layer^36^. The result of secondary growth is the division of coordination cells in the meristem region of xylem and the differentiation of progeny cells in endodermis and wood tissue^37^. The results of microarray analysis showed that there was a good correlation between the transcriptional level of genes and the function in cell division and differentiation at specific stages of wood development^12,38^. It indicated that *ARK1* was vital to regulating poplar growth. The current study selected 27 DEGs involved in poplar growth by analysing the DEGs in the pathways of plant hormone signal transduction (Ko04075), phenylpropanoid biosynthesis (Ko00940), cell tip growth (GO:0009932) and regulation of meristem growth (GO:0010075). These genes included coding proteins, transcription factors and protein kinases, which were related to plant growth and development and lignin regulation. It illustrated that *ARK1* was also crucial for regulating poplar growth.

This study found that the enzymes related to lignin synthesis in the phenylpropanoid pathway were down-regulated. Lignin is one of the three main chemical components of wood (lignin, cellulose and hemicellulose) and has essential biological functions in plants^39,40^. Lignin limits the development of the paper industry due to environmental pollution and the need for a large amount of energy for wood production in the process of paper-making. The reduction of lignin content of trees can not only improve the economic and environmental benefits of the pulp and paper industry but also promote the decomposition of lignocellulose and improve the conversion efficiency of sugar^41^. The changes of lignin content and composition had no adverse effect on the growth of transgenic plants but increased the biomass of transgenic plants, such as stem diameter, plant height and internode length. Zhou *et al*. (2018) found that when the lignin content of C3H and HCT transgenic hybrid poplars (*P. alba* × *P. glandulosa* ‘84 K’) decreased, the phenotype of plants showed abnormal growth in height, diameter, and so on^42^. Su *et al*. (2019) found that when the content of lignin reduced, the content of lignin deposited in the cell wall decreased, which easily led to the abnormal phenotype of tissue culture seedling^43^. This study proposes that the abnormal phenotype of *ARK1* transgenic poplar in node spacing, stem diameter, petiole length, leaf width, leaf length and seedling height is due to the down-regulation of the enzymes related to lignin synthesis.

Chromatin immunoprecipitation sequencing (ChIP-seq) technology was employed to identify *ARK1* binding loci. Findings showed that *ARK1* is a vital transcription factor of the vascular cambium and cell differentiation regulation in Populus^12^. This study also found that *ARK1* is a key regulator of cell differentiation in Populus. However, Liu *et al*. (2015) did not report a relationship between the expression of *ARK1* and the expression of enzymes related to lignin synthesis^12^. Our results also showed that when the expression level of genes related to the lignin content of plants reduced, the plants would grow abnormally in terms of height and diameter. Groover *et al*. (2006) reported a similar phenotype of *ARK1* transgenic poplar after analysing transcriptome data (using microarray). However, more cell-wall associated GO terms were found in Groover and colleagues’ study. The current study found lignin biosynthesis genes to be mostly down-regulated in *ARK1*-overexpressing lines, whereas Groover *et al*. (2006) found 35S::*ARK1* trees to have increased lignin, which is paradoxical. Of course, the reduction in lignin gene expression was only based on two genes; thus, this finding requires further support via future studies.

## Conclusions

The *ARK1* was transformed into hybrid poplar ‘717’. PCR detection showed that the positive rate was 31.43%. There were significant differences in node spacing, stem diameter, petiole length, leaf width, leaf length and seedling height between transgenic and non-transgenic seedlings. The stem segments of transgenic ‘717’ hybrid poplar seedlings were slender, fasciculated and multi-branched. The leaves were slender, and some leaves were not fully developed.

Twenty-seven DEGs involved in poplar growth and development were screened out, including proteins, transcription factors and protein kinases. The up-regulated DEGs were mainly positive regulatory genes, while the down-regulated DEGs were mainly negative regulatory genes. The *ARK1* gene had a positive regulatory effect on plant growth, and the gene’s coding enzymes related to lignin synthesis were down-regulated.

## Data Availability

RNA-seq data were presented at the Genome Sequence Archive of National Genomics Data Center, Beijing Institute of Genomics (accession number CRA002209).
